# Resource Availability Shaped the Female‐Biased Floral Sex Allocation With Plant Size in Andromonoecious *Capparis spinosa* in Desert

**DOI:** 10.1002/ece3.74605

**Published:** 2026-11-02

**Authors:** Yu‐Jie Cai, Yang Lu, Aysajan Abdusalam

**Affiliations:** ^1^ College of Life and Geographic Sciences Kashi University Kashi Xinjiang China; ^2^ Key Laboratory of Biological Resources and Ecology of Pamirs Plateau in Xinjiang Uygur Autonomous Region Kashi University Kashi Xinjiang China

**Keywords:** Andromonoecy, *Capparis spinosa*, floral sex allocation, phenotypic plasticity, resource availability, size‐dependent

## Abstract

Although classic models of size‐dependent sex allocation (SDS) generally predict that among animal‐pollinated plants, female allocation increases with individual size, male‐and female‐biased allocation patterns have also been reported. We hypothesized that *Capparis spinosa* growing in resource‐poor desert habitats would exhibit a female‐biased sex allocation with increasing plant size, and that this pattern would be directly regulated by resource availability, because female function is generally more resource‐demanding to verify this conjecture. We examined SDS patterns at the flowering stage in the andromonoecious desert plant *C. spinosa* and assessed the role of resource availability in shaping these patterns through resource supplementation and pruning treatments. The results indicated that male plants with a low biomass occurred at low frequencies (0.021–0.051) in seven of the nine assessed populations. Among andromonoecious individuals across the nine populations, with increasing plant size, we recorded increases in the numbers of male and hermaphroditic flowers, the proportion of hermaphroditic flowers, and values of Lloyd's phenotypic gender index. These findings indicate a strong female‐biased sex allocation in *C. spinosa* plants of larger size. In addition, 2‐year resource manipulation experiments revealed that both the supplementation of water and nutrients and pruning treatments significantly enhanced the proportion of hermaphroditic flowers, thereby indicating that floral sex allocation is directly regulated by resource availability. Our findings in this study highlight the role of resource availability in shaping size‐dependent sex allocation patterns, particularly for plants in resource‐poor desert habitats, and sheds light on the evolution of sexual systems in plants.

## Graphical abstract

This study investigates how resource availability drives size‐dependent sex allocation in the andromonoecious desert plant *Capparis spinosa* . Through field surveys across nine populations and two‐year resource manipulation experiments (water–nutrient supplementation and pruning), we demonstrate that larger plants produce a higher proportion of hermaphroditic flowers only when resources are sufficient, revealing that female‐biased allocation is dynamically regulated by resource availability rather than being a fixed size‐dependent trait.

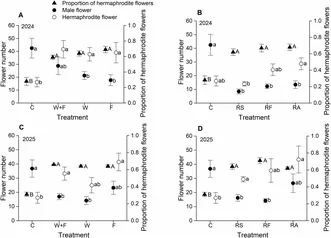

## 1 Introduction

Sex allocation constitutes a pivotal topic within the disciplines of plant reproductive ecology and evolutionary biology, specifically pertaining to the strategies whereby plants optimally allocate limited resources between male and female functions to maximize their fitness (Charnov 1982; Chen and Pannell 2025a; Gerchen et al. 2026). As a significant extension of sex allocation theory, the concept of size‐dependent sex allocation (SDS) posits that smaller individuals generally invest in the less resource‐intensive sex, typically males, whereas larger individuals, endowed with more substantial maternal resources, tend to be characterized by a greater female‐orientation investment (Lloyd and Bawa 1984; Vega‐Frutis et al. 2014; Chen and Pannell 2025a). Plants adjust their investment in male and female functions in accordance with individual size, which serves as a core indicator of resource reserves (Lloyd and Bawa 1984; Zhang et al. 2014). This adjustment pattern is determined by the rates at which male and female fitness gain curves saturate, along with habitat selection pressures and modes of pollination (Klinkhamer and De Jong 1997; Friedman and Barrett 2011; Nakahara et al. 2018; Chen and Pannell 2025b). Extensive research on size‐dependent sex allocation has contributed to enhancing our understanding of the principles governing resource allocation between vegetative and reproductive growth, as well as the evolutionary mechanisms associated with plant reproductive systems (Cao and Kudo 2008; Barrett 2013; Zhang et al. 2014; Nakahara et al. 2018; Coelho et al. 2021). However, when specifically examining floral sex allocation during the flowering stage, using metrics such as the pollen/ovule ratio or the proportions of male and female flowers, the relationship between size and sex allocation is found to differ among species, pollination modes, and resource conditions. For example, under conditions of resource limitation, plants are typically characterized by a male‐biased sex allocation (Liao et al. 2006; Zhao et al. 2015; Nepal et al. 2023). However, in some wind‐pollinated species, such as *Ambrosia artemisifolia* and *Pennisetum typhoides* , sex allocation may be male‐biased in larger plants because of the direct height effects on pollen dispersal (Dajoz and Sandmeier 1997; Nakahara et al. 2018). The relationship between size and sex allocation has also been reported in numerous insect‐pollinated plant species, among which a female‐biased sex allocation is commonly observed in larger plants (Biernaskie and Elle 2005; Cao and Kudo 2008; Xiong et al. 2015).

Accumulating evidence indicates that resource availability is a key factor regulating differences in size‐dependent sex allocation in andromonoecious individuals (Cuevas and Polito 2004; Calviño and Galetto 2010). Because plants of different sizes differ in their capacity to acquire resources, larger individuals allocate more resources to reproduction, with plant size serving as an indicator of resource reserves that only indirectly affects the amount of available resources (Klinkhamer and De Jong 1997; Klinkhamer et al. 1997; Zhang and Tan 2012; Coelho et al. 2021). In resource‐rich environments, plants are generally larger and allocate a greater proportion of resources to female function, thus shifting the population towards a female‐biased direction; whereas under stressful conditions, plants are smaller and allocate more to male function, resulting in a male‐biased direction (Solomon 1985; Zhang 2004; Coelho et al. 2021). For example, Quagliotti (1967) reported that *Daucus carota* showed significantly lower male fertility under high temperatures, while low temperatures favored hermaphroditic flowers. In *Solanum carolinense* , shading and drought stress increased the proportion of male flowers, thereby maintaining overall plant fitness (Solomon 1985). Moreover, in *Solanum lycocarpum* , the production of male flowers was more pronounced during the rainy season and decreased during the dry season (Marcelo et al. 2021), whereas in the perennial species *Lilium concolor* var. *megalanthum*, hermaphroditic flowers tended to develop under high‐nutrient conditions, while plants under low‐nutrient conditions generally either produced male flowers or remained non‐flowering (Chen et al. 2023). Therefore, it is important to report the sex allocation strategies of andromonoecious plants growing in extreme habitats such as deserts. However, most existing evidence is derived from comparisons across different habitats or years, which are essentially correlative inferences, and the direct regulatory role of resource availability on sex allocation still lacks more manipulative experimental verification.

Resource availability is commonly divided into exogenous and endogenous resources. Exogenous resources refer to those acquired from the external environment through root uptake or leaf capture, primarily including soil mineral nutrients (nitrogen, phosphorus, potassium, etc.), water, and light. Exogenous resources influence the total resources available for floral development by altering a plant's resource acquisition capacity (Bloom et al. 1985; Bazzaz and Grace 1997; Sato 2004). For example, supplemental fertilization significantly increased the proportion of hermaphroditic flowers, fruit set, and individual fruit dry weight in olive ( *Olea europaea* ), indicating that female reproductive function is directly limited by nutritional resources (Zhu et al. 2015). In pomegranate ( *Punica granatum* ), higher levels of nitrogen and phosphorus in the first year increased the proportion of hermaphroditic flowers (Lazare et al. 2020). Endogenous resources, in contrast, refer to resources that have already been synthesized or stored within the plant and are directly available for floral organs, as well as the plant's structural resources (e.g., photosynthetic area, storage organs, bud number). Endogenous resource manipulation does not alter environmental supply, but rather simulates resource competition by modifying the plant's internal resource allocation pattern or source–sink relationships (Wiley et al. 2013). For instance, in the andromonoecious wild olive tree *Olea europaea* var. *sylvestris*, removing half of the inflorescences (thereby increasing resources allocating to sexual reproduction) raises the probability of hermaphroditic flower development, whereas in contrast, removing half of the leaves (reducing resource amount from photosynthesis) reduces the probability of hermaphroditic flowers developing at different positions, albeit weakly (Granado‐Yela et al. 2017). These studies indicate that resource availability can directly regulate floral sex allocation; however, manipulative experiments directly testing this relationship remain limited in desert plants.

*Capparis spinosa* , a typical andromonoecious plant with functional male and hermaphroditic flowers, is widely distributed in arid and semi‐arid desert regions, in which it shows strong environmental adaptability and phenotypic plasticity (Zhang and Tan 2009; Afzali et al. 2023), traits that make it ideally suited to evaluation of the effects of resource availability on size‐dependent sex allocation in desert plants. At present, however, there has been little experimental verification of the association between individual size and floral sex allocation in this plant, or whether the observed trend of larger individuals being more female‐biased is promoted by resource limitation.

With a view towards quantifying floral sex allocation with respect to plant size and evaluating the influence of resource availability on SDS patterns under conditions of extreme resource limitation, as the research system, we focused on the desert andromonoecious plant *Capparis spinosa* growing in a region near the Taklamakan Desert in southern Xinjiang, China. By integrating field investigations and resource‐controlled experiments, we aim to address the following scientific questions. (1) Are larger individuals of *C. spinosa* growing in an arid environment under resource‐limited conditions characterized by a female‐biased floral sex allocation? (2) Will exogenous resource supplementation (watering and fertilization) or endogenous resource re‐allocation (pruning) directly alter its floral sex allocation patterns? We accordingly attempted to obtain empirical evidence for the hypothesis that “resource availability drives female‐biased SDS in andromonoecious plants” and provide a new perspective on the evolutionary adaptation of plant sex allocation in extreme habitats.

## 2 Materials and Methods

### 2.1 Research Materials and Study Site

*Capparis spinosa* L. is a perennial woody vine (small semi‐shrub) in the family Capparaceae, which is distributed in regions of the Mediterranean, Central Asia, and West Asia. In China, the plant is mainly found in Xinjiang, Gansu, and Tibet (Chedraoui et al. 2017). It has a well‐developed root system and can thus be used to facilitate soil and water conservation. The plant typically grows as a prostrate shrub bearing numerous branches, often drooping or climbing, with solitary flowers borne in leaf axils. Hermaphroditic flowers produce fertile pollen and ovaries that can develop normally into fruits. Although functional male flowers also produce fertile pollen, the considerably smaller ovaries fail to complete development into fruits (Zhang and Tan 2009). The peak flowering period of *C. spinosa* differs according to local climatic conditions.

This study of size‐dependent sex allocation was carried out among nine natural populations of *C. spinosa* (Table 1), all of which are distributed in the vicinity of the Taklamakan Desert in southern Xinjiang, where the average altitude exceeds 1000 m. The region is characterized by a typical temperate desert climate with scant precipitation. Whereas the annual rainfall averages less than 50 mm, the annual rate of evaporation can reach as high as 3800.0 mm, leading to extreme aridity. The soil types mainly comprise silt. Controlled experiments on resource manipulation were conducted in the population located in Gédaliáng Township, southern Xinjiang (39°55′28.50′′N, 76°57′34.06′′E; altitude 1329 m).

**TABLE 1:** Geographical information, climatic conditions, and sexual structure of nine natural populations of *Capparis spinosa* (Mean ± SE).

Population	Location	Altitude (m)	Mean monthly temperature (°C)	Mean monthly rainfall (mm)	Number of andromonoecious plants	Number of male plants	Proportion of male plants	Aboveground biomass (g)	Number of male flowers	Number of hermaphroditic flowers
Jwz	41°18′13.7″N	1270	11.56	9.07	172	4	0.023	77.88 ± 7.72	86.86 ± 9.61	29.75 ± 4.30
	79°36′77.2″E									
Bjx	41°18′13.14″N	1368	11.56	9.07	228	12	0.050	118.07 ± 14.19	83.86 ± 11.02	38.19 ± 6.48
	79°36′77.9″E									
Ly	41°19′91.1″N	1388	12.37	5.16	148	8	0.051	69.74 ± 6.95	42.87 ± 6.43	34.33 ± 3.87
	79°23′96.8″E									
Smy	41°52′85.6″N	1478	14.64	11.77	295	0	0.000	83.97 ± 9.38	82.97 ± 10.08	48.10 ± 7.01
	79°80′28.3″E									
Ahq	41°18′13.13″N	1501	12.37	5.16	164	4	0.024	74.28 ± 8.01	43.21 ± 5.05	21.60 ± 3.12
	79°36′77.8″E									
Hbq	41°27′56.4″N	1564	14.21	7.69	147	6	0.039	57.03 ± 6.22	82.97 ± 10.08	33.36 ± 5.30
	80°23′68.3″E									
Wq	39°60′16.7″N	1899	9.56	28.53	376	8	0.021	169.45 ± 16.34	126.88 ± 15.83	76.00 ± 14.40
	75°06′84.6″E									
Akt	38°77′70.8″N	2209	13.66	6.17	400	0	0.000	169.42 ± 16.91	179.30 ± 26.18	85.24 ± 12.38
	75°31′75.1″E									
Tx	37°31′76.6″N	2384	3.27	11.13	200	8	0.039	88.37 ± 8.40	78.90 ± 8.70	30.34 ± 4.79
	75°94′89.3″E									

*Note:* The study populations were as follows: JWZ, BJX, and LY (Wushi); SMY and HBQ (Wensu); AHQ (Aheqi); WQ (Wuqia); AKT (Aketao); and TX (Taxian). The proportion of male plants was calculated as the ratio of the number of male plants to the total number of plants.

### 2.2 Research Methods

#### 2.2.1 Individual Size Measurements

We conducted detailed surveys among nine natural populations of *C. spinosa* in southern Xinjiang, in each of which the sexual system types (male and andromonoecious) of 200–400 plants were determined. In April 2024, in each of the nine populations, 50–60 *C. spinosa* plants of differing size were marked and enclosed within nylon mesh bags (mesh size: 12 mm) to prevent flower loss caused by wind or rain prior to flower counting; the bags were removed immediately after flower counting to allow normal fruit development. Given the clonal growth habit of this species, marked individuals were maintained at distances greater than 10 m from each other. After the flowering period, the above‐ground parts of plants were harvested, dried in an oven at 80°C, and weighed, with the values thus obtained serving as an indicator of individual plant size.

#### 2.2.2 Floral Sex Allocation and Sexual Phenotype at the Population Level

To quantify individual floral sex allocation, during the peak flowering period, we recorded the numbers of male and hermaphroditic flowers per plant among marked individuals in the nine *C. spinosa* populations, and thereby obtained values for the total number of flowers (the sum of male and hermaphroditic flowers) per plant. As indices of floral sex allocation, we used values of the phenotypic gender index (Gi), which shows a high correlation with the percentage of hermaphroditic flowers.

The phenotypic gender of *C. spinosa* plants was quantified at the population level using Lloyd's formula (Lloyd 1980) as follows:

$$\mathrm{Gi}=\frac{\mathrm{oi}}{\left(\mathrm{oi}+\mathrm{pi}E\right)},$$

where $E=\frac{\sum \mathrm{oi}}{\sum \mathrm{pi}}$; oi represents the female investment of plant i, measured as the number of ovule‐producing flowers (hermaphrodite flowers); pi denotes the male investment of plant i, measured as the number of pollen‐producing flowers (hermaphrodite flowers and male flowers); and E is the ratio of the total female investment to the total male investment among all individuals in the population. The value of Gi ranges from 0 (plants producing only pollen) to 1 (plants producing only ovules), with higher Gi values indicating a more female‐biased phenotypic gender. This index facilitates a standardization of the sex bias at the population level, thereby facilitating comparisons among individuals and populations.

#### 2.2.3 Resource Supplementation and Pruning

To evaluate the influence of resource availability on the allocation to male and hermaphroditic flowers in *C. spinosa* , 70 healthy plants of approximately similar size were selected for a resource manipulation experiment during the 2024 and 2025 growing seasons. Ten plants were randomly assigned to each treatment and we compared floral sex allocation in response to exogenous resource supplementation (watering and fertilization) and endogenous resource reallocation (pruning). The resource supplementation treatments were conducted from mid‐to‐late April until late May, while the pruning treatments were applied as a single event at the initial formation of flower buds in mid‐to‐late April. During the peak flowering period in late May, the numbers of male and hermaphroditic flowers per plant were counted for each treatment.

Supplementation was assessed by performing the following four treatments: (1) Control (C): no watering or fertilization; (2) watering and fertilization (W + F): commencing from the initial formation of flower buds in mid‐to‐late April, plants were irrigated with 2 L of canal water in the morning once weekly until late May, with N‐P‐K (15–15‐15) compound fertilizer being applied during the mid‐April growth period (fertilizer solution was prepared according to the dilution ratio specified on the label: 4.16 g/2000 mL); (3) watering (W): commencing from the initial formation of flower buds in mid‐to‐late April, plants were irrigated daily in the morning once weekly with 2 L of canal water until late May; and (4) fertilization (F): N‐P‐K (15–15‐15) compound fertilizer (fertilizer solution was prepared according to the dilution ratio specified on the label: 4.16 g/2000 mL) was applied during the mid‐April growth period.

Endogenous resource re‐allocation involved the following four treatments: (1) Control (C): no bud removal or branch pruning; (2) removal of half the number of stolons (RS); (3) removal of half the number of floral buds (RF): flower buds on the basal part of each stolon were removed, with the remainder of the plant left intact; and (4) removal of the 10‐cm tip of branches (RA); pruning of approximately 10 cm of the terminal portion of each branch. The same set of control plants was used for both the supplementation and pruning experiments.

### 2.3 Statistical Analysis

All statistical analyses were performed using SPSS 31.0 (SPSS Inc.), and figures were created using OriginPro 2025. Analyses were tailored to the data type and distribution of each response variable as follows: (1) Comparison between male and andromonoecious plants: We constructed generalized linear models (GLMs) with plant sexual system type as the explanatory variable (fixed factor) and aboveground biomass and the number of male flowers as separate response variables. Aboveground biomass was modeled with a Gamma distribution and a log link function, and the number of male flowers was modeled with a negative binomial distribution and a log link function. (2) Association between plant size and floral traits: Because the data did not meet the assumptions of normality and homogeneity of variance, we used Spearman rank correlation analysis to examine the relationships between aboveground biomass and the numbers of male/hermaphroditic flowers and Gi values across the nine populations. For correlated variables, we then performed simple linear regression analysis. (3) Effects of resource treatments on floral allocation: We modeled the 2024 and 2025 datasets independently. For flower counts, we constructed GLMs with a Poisson distribution and a log link function, with the numbers of male and hermaphroditic flowers as response variables and treatment type (fixed factor) as the explanatory variable. For the proportion of hermaphroditic flowers, we constructed GLMs with a binomial distribution and a logit link function, with the number of hermaphroditic flowers as the response variable and the total number of flowers (hermaphroditic + male) included as the binomial denominator; treatment type (fixed factor) was the explanatory variable.

1. Comparison between male and andromonoecious plants: We constructed generalized linear models (GLMs) with plant sexual system type as the explanatory variable (fixed factor) and aboveground biomass and the number of male flowers as separate response variables. Aboveground biomass was modeled with a Gamma distribution and a log link function, and the number of male flowers was modeled with a negative binomial distribution and a log link function.
2. Association between plant size and floral traits: Because the data did not meet the assumptions of normality and homogeneity of variance, we used Spearman rank correlation analysis to examine the relationships between aboveground biomass and the numbers of male/hermaphroditic flowers and Gi values across the nine populations. For correlated variables, we then performed simple linear regression analysis.
3. Effects of resource treatments on floral allocation: We modeled the 2024 and 2025 datasets independently. For flower counts, we constructed GLMs with a Poisson distribution and a log link function, with the numbers of male and hermaphroditic flowers as response variables and treatment type (fixed factor) as the explanatory variable. For the proportion of hermaphroditic flowers, we constructed GLMs with a binomial distribution and a logit link function, with the number of hermaphroditic flowers as the response variable and the total number of flowers (hermaphroditic + male) included as the binomial denominator; treatment type (fixed factor) was the explanatory variable.

All models used Huber‐White robust standard errors for parameter estimation. For models with significant treatment effects, we applied Bonferroni correction for post hoc pairwise comparisons. The significance level for all tests was set at *α* = 0.05.

## 3 Results

### 3.1 Variations in Sexual Expression Among Populations

Male plants were identified in seven of the nine populations, albeit at low frequencies, ranging from 0.021 to 0.051, with the majority of *C. spinosa* plants being identified as andromonoecious, bearing both male and hermaphroditic flowers. In addition to contrasting abundance, male and andromonoecious plants were found to be characterized by differences regarding biomass and flower number (Table 1, Figure 1). Specifically, the above‐ground biomass of male plants (15.41 ± 3.35 g) was significantly lower than the 103.00 ± 3.74 g of andromonoecious plants (Wald *χ* ^2^ = 74.297, *p* < 0.001). Moreover, the number of male flowers on male plants (17.08 ± 4.39) was significantly lower than that of andromonoecious plants (88.87 ± 4.35) (Wald *χ* ^2^ = 39.775, *p* < 0.001, Figure 1).

**FIGURE 1:**
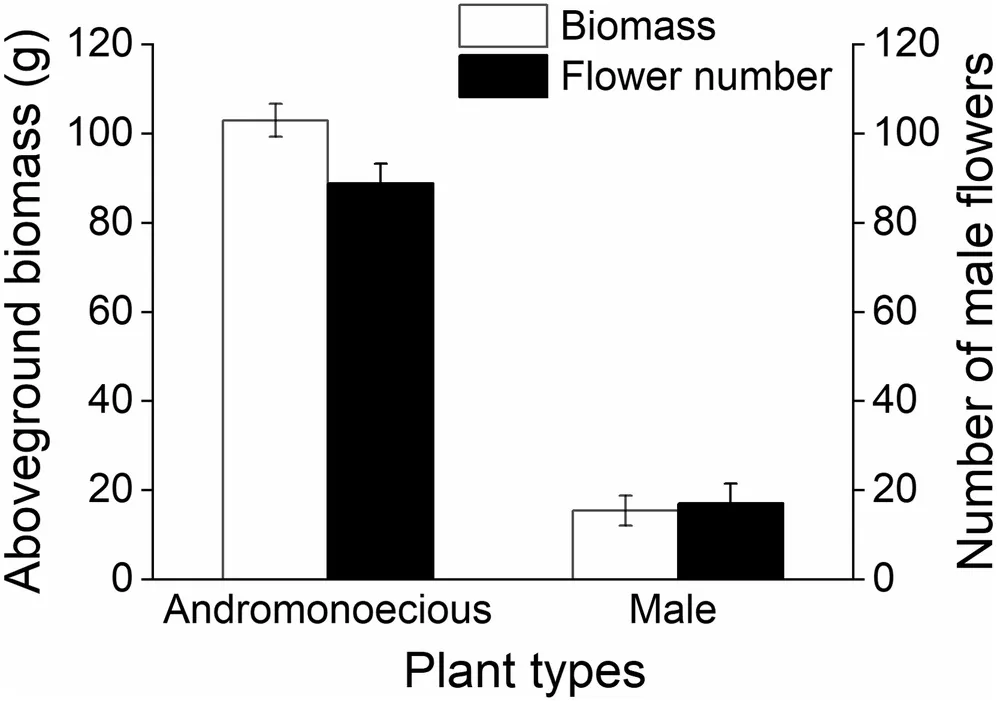
Comparison of above‐ground biomass and number of male flowers of andromonoecious and male plants (Mean ± SE).

### 3.2 Floral Investment and Allocation in Relation to Plant Size

The numbers of both male and hermaphroditic flowers showed significant positive correlations with plant size (above‐ground biomass) and increased lineally among the nine populations (Figure 2). Femaleness, calculated based on phenotypic gender (Gi value), was positively correlated with plant size in all nine populations (Table S1) and increased lineally in five (JWZ, BJX, SMY, AHQ, and WQ; Figure 3). When the values for all nine populations were pooled, we detected an increase in investment in both male (*N* = 442, *R* ^2^ = 0.56, *p* < 0.001) and hermaphroditic (*N* = 442, *R* ^2^ = 0.60, *p* < 0.001) flowers with increasing plant size, whereas the phenotypic gender index (Gi) significantly increased with increasing plant size (*N* = 442, *R* ^2^ = 0.026, *p* < 0.001; Figure 3)., indicating a more female‐biased sex allocation in larger plants.

**FIGURE 2:**
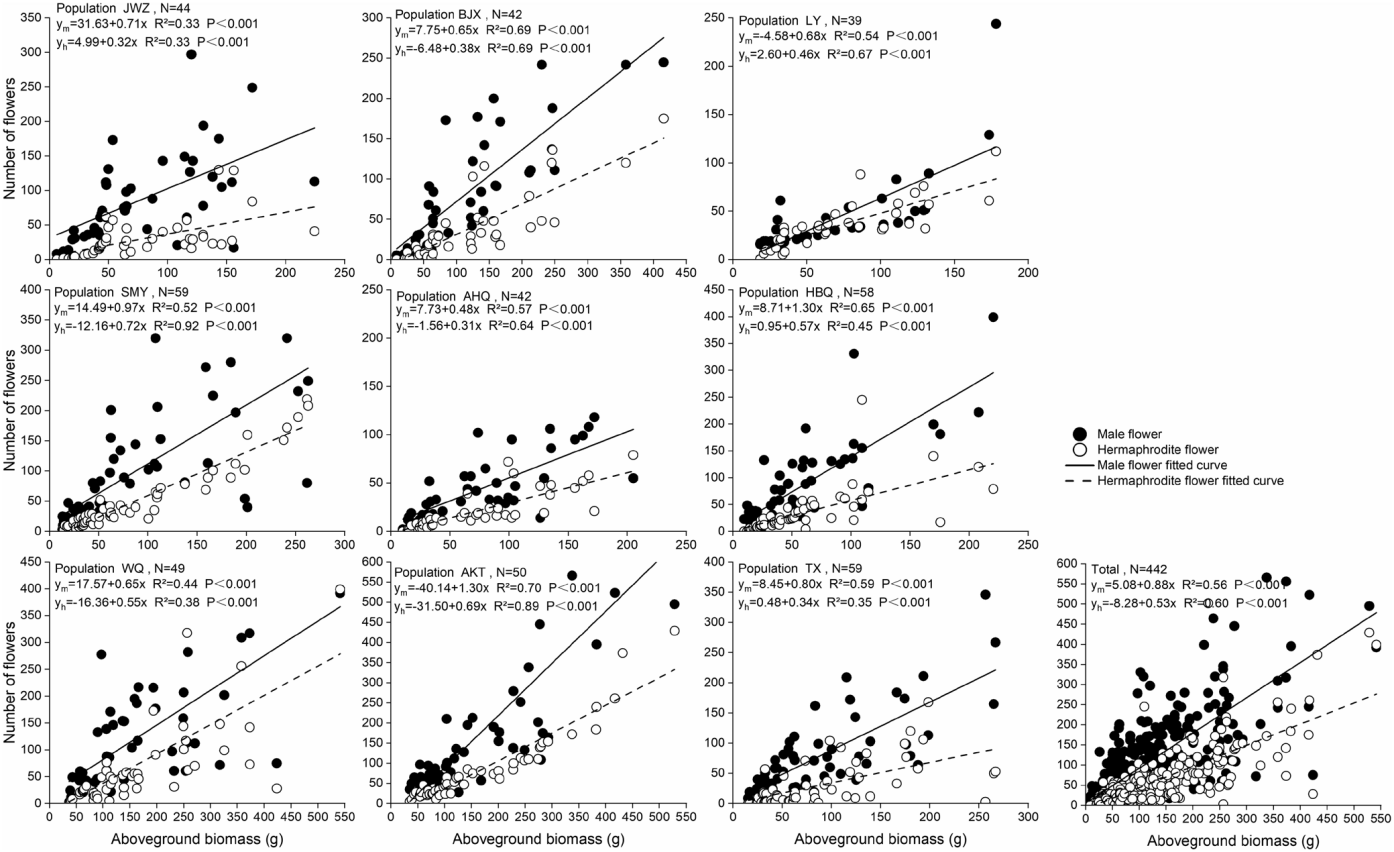
Linear regressions of male/hermaphroditic flower production on plant size in *Capparis spinosa* .

**FIGURE 3:**
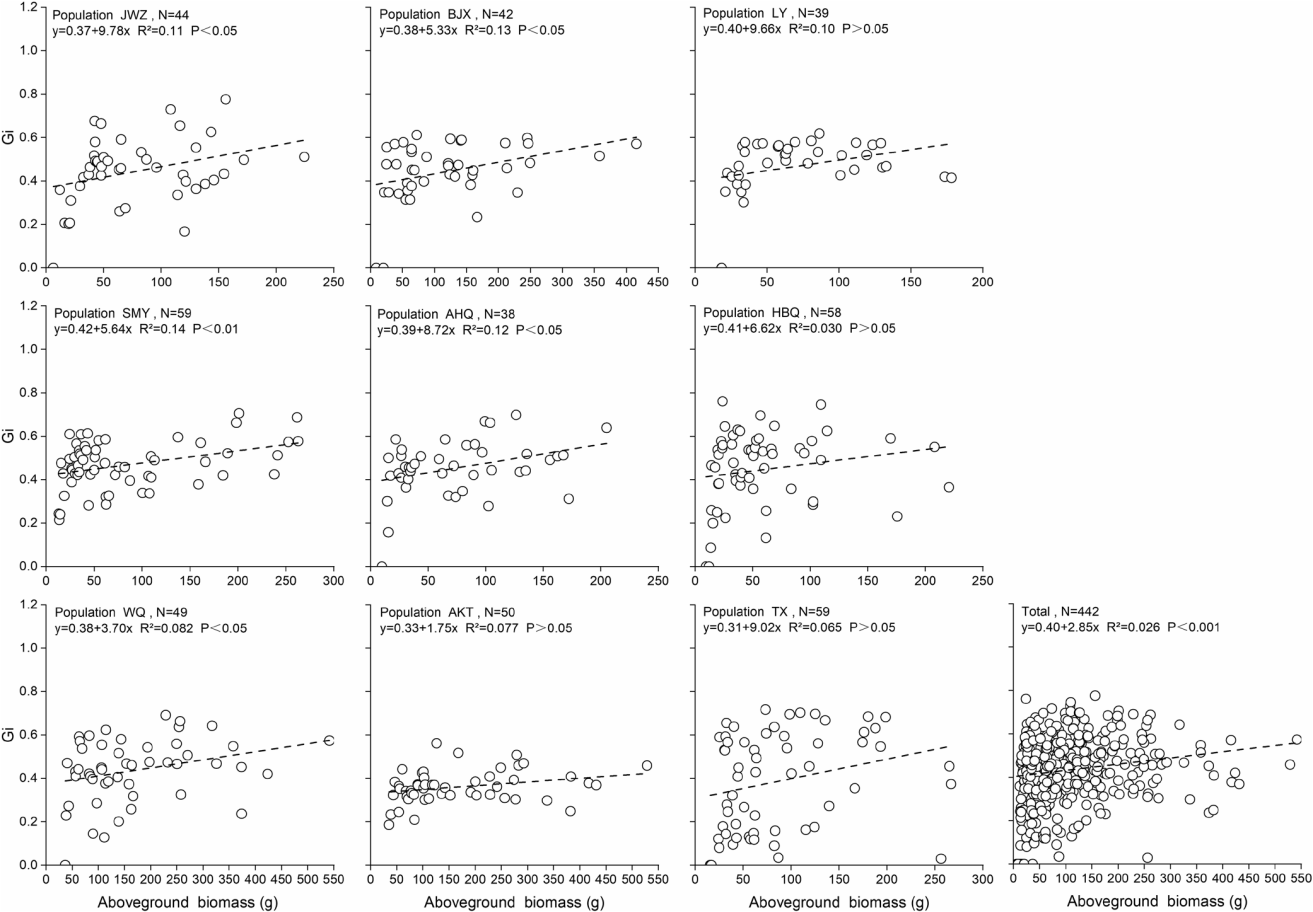
Linear regression of Gi values on plant size in *Capparis spinosa* .

### 3.3 Effects of Resource Availability on Floral Allocation

In both 2024 and 2025, we obtained evidence to indicate that resource allocation to both male and hermaphroditic flowers was significantly constrained by resource availability. In response to increases in the levels of watering and fertilization, we recorded significant reductions in the number of male flowers (solid circles: Figure 4A, Wald *χ* ^2^ = 12.302, *p* < 0.01; Figure 4C, Wald *χ* ^2^ = 18.031, *p* < 0.001), increases in the number of hermaphroditic flowers (open circles: Figure 4A, Wald *χ* ^2^ = 13.382, *p* < 0.01; Figure 4C, Wald *χ* ^2^ = 12.272, *p* < 0.01), and an increase in the proportion of hermaphroditic flowers (triangular symbols: Figure 4A, Wald *χ* ^2^ = 33.463, *p* < 0.001; Figure 4C, Wald *χ* ^2^ = 143.438, *p* < 0.001). Similarly, in response to an increase in the resources saved from other organs (through pruning), there were reductions in the number of male flowers (solid circles: Figure 4B, Wald *χ* ^2^ = 42.628, *p* < 0.001; Figure 4D, Wald *χ* ^2^ = 27.009, *p* < 0.001), along with increases in the number of hermaphroditic flowers (open circles: Figure 4B, Wald *χ* ^2^ = 17.754, *p* < 0.001; Figure 4D, Wald *χ* ^2^ = 9.996, *p* < 0.05) and the proportion of hermaphroditic flowers (triangular symbols: Figure 4B, Wald *χ* ^2^ = 34.061, *p* < 0.001; Figure 4D, Wald *χ* ^2^ = 106.163, *p* < 0.001).

**FIGURE 4:**
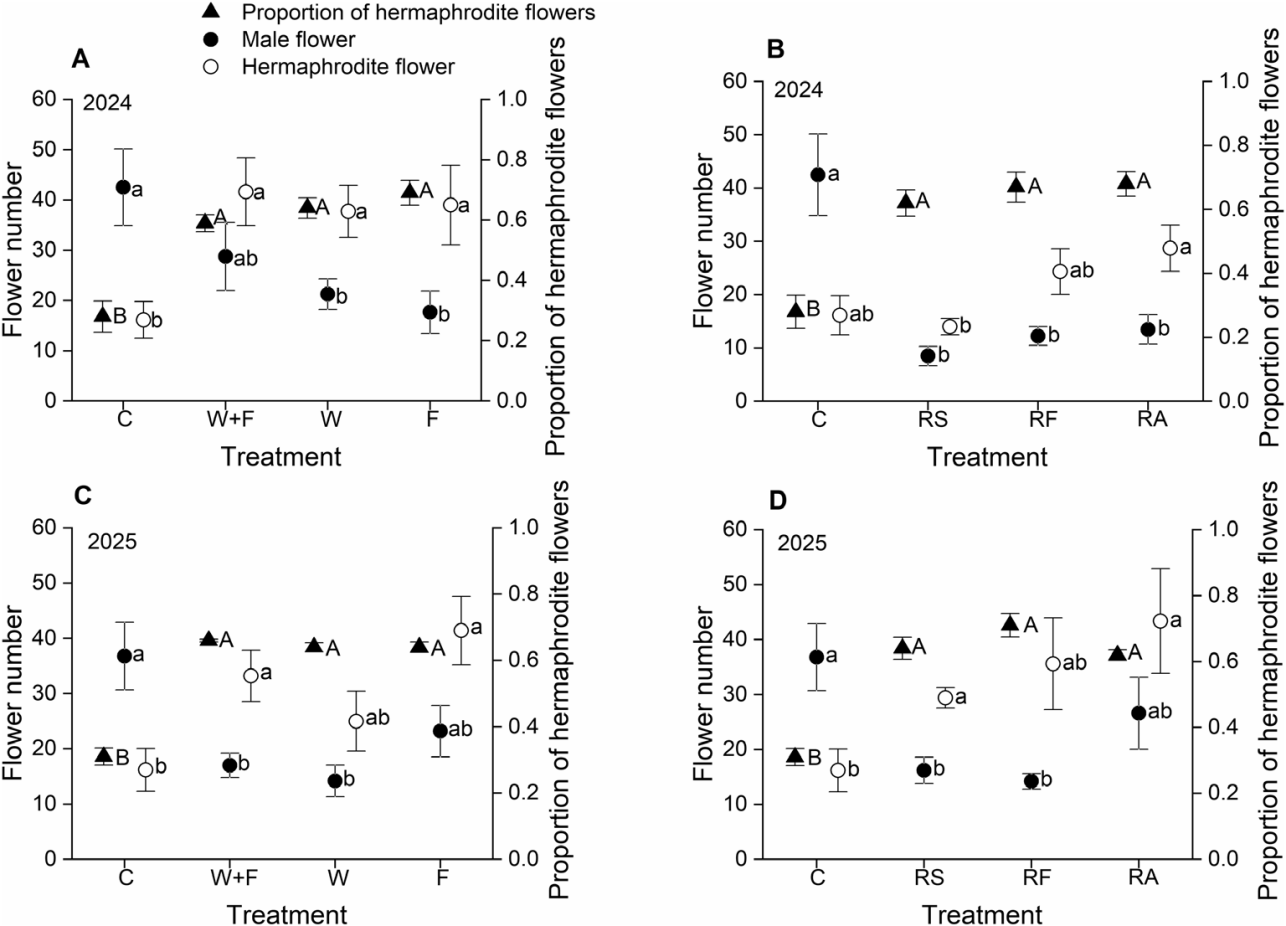
Effects of watering–fertilization and pruning treatments on the number of male flowers, number of hermaphroditic flowers, and proportion of hermaphroditic flowers in *Capparis spinosa* (Mean ± SE). (A, C) Influence of watering and fertilization on the number of flowers and the proportion of hermaphroditic flowers in *C. spinosa* . (B, D) Influence of different pruning treatments on the number of flowers and the proportion of hermaphroditic flowers in *C. spinosa* . C, No pruning; C, No watering or fertilization; F, Fertilization; RA, Removal of the terminal 10 cm portion of branches; RF, Removal of half the number of floral buds; RS, Removal of half the number of stolons; W + F, Watering and fertilization; W, Watering. Different uppercase letters indicate significant differences in the proportion of hermaphroditic flowers among treatments; different lowercase letters denote significant differences in the numbers of male and hermaphroditic flowers among treatments.

## 4 Discussion

In this study, we systematically investigated the size dependence of sex allocation during the flowering phase of the desert plant *Capparis spinosa* and its response to resource availability. In all nine of the assessed populations, we observed a pronounced female‐biased floral sex allocation with increasing plant size (Figures 2 and 3). By experimentally manipulating resource availability, we found that regardless of whether resources were increased via exogenous supplementation based on watering and fertilization or endogenously in response to pruning, there were significant increases in the proportion of hermaphroditic flowers (Figure 4). These findings accordingly provide evidence to indicate that SDS patterns are regulated by resource availability.

Size‐dependent sex allocation has been suggested to provide insights into the evolution of plant sexual systems, as has been described for numerous taxa (Peruzzi et al. 2012; Shwe et al. 2020; Coelho et al. 2021; Zhang et al. 2025), with both male‐biased and female‐biased sex allocation having been reported (Andrieu et al. 2007; Friedman and Barrett 2011; Wang et al. 2019; Chen and Pannell 2025a). In all nine of the assessed *C. spinosa* populations, we observed increases in the proportions of both male and hermaphroditic flowers with increasing above‐ground biomass (Figure 2), thereby indicating that plant size has a notable influence on sex investment. We detected a consistent female‐biased floral sex allocation among the nine populations, whereas in each of the populations and the total study population, there were increases in Lloyd's phenotypic gender index with increases in plant size (Table S1, Figure 3). The theoretical model predicted that plants of a particularly small size (less than a specified threshold) would be exclusively characterized by male function and, indeed, we found male plants occurring at low frequency in four populations (Table 1), which were smaller in biomass and produced fewer flowers compared with counterpart andromonoecious plants (Figure 1). This pattern of size‐dependent sex allocation, which inclines towards femaleness in larger plants during the flowering stage, is consistent with the findings reported for *Lilium apertum* (Zhang et al. 2014), *Solanum lycocarpum* (Coelho et al. 2021), and several species within the family Apiaceae (Zhang and Tan 2012; Kudo and Shibata 2021). Nevertheless, such size‐dependent sex allocation is not invariably detected among species. For example, Nakahara et al. (2018) have reported an inverse pattern for *Ambrosia artemisiifolia* , in which larger individuals produce a larger number of male flowers. In contrast, no significant correlation between plant size and flower sex ratios has been detected in *Solanum houstonii* (Zapata Carbonell 2019).

This size‐dependent sex allocation, biased towards males or females, may stem from how “individual size” is measured and its associated ecological implications, which can be illustrated by studies in which plant height is utilized as a surrogate for size. Taller individuals generally augment male function during the pollination process, and in both anemophilous and entomophilous plant species, an increase in height can contribute to increasing the frequency of pollinator visitations and pollen dispersal, thereby enhancing male fitness, leading to a “larger individuals are more male” pattern (Ge et al. 2012; Nakahara et al. 2018). Notably, in this study, we used above‐ground biomass as an index of individual size, a metric widely utilized in plant ecology that effectively reflects nutritional status and resource acquisition capacity (Weiner et al. 2009). Consequently, the pollination benefits conferred by height are largely inapplicable in this context.

A further plausible factor accounting for the inconsistent findings for size‐dependent sex allocation is how SDS patterns are constrained by available resources. Sex allocation theory posits that when resources are abundant, individuals should allocate a larger proportion of these resources to the more resource‐demanding female functions (Lloyd and Bawa 1984). Accordingly, resource manipulation experiments are necessary to identify the potential mechanisms associated with SDS patterns. In this regard, we found that compared with male‐associated functions, the female functions are more resource‐demanding, as evidenced by our observations that male individuals were smaller in biomass than andromonoecious individuals in *C. spinosa* populations (Figure 1). In addition, we conducted resource supplementation experiments to assess whether the exogenous supplementation of resources could contribute to a more female‐biased allocation and, indeed, found that supplementation with water and fertilizer promoted significant increases in the numbers and proportions of hermaphroditic flowers, whilst contributing to reductions in the number of male flowers (Figure 4A,C). Furthermore, pruning treatments were performed to evaluate whether the resources accumulated by other organs could undergo re‐allocation and augment female allocation. In response to these treatments, namely, removal of half the number of stolons and floral buds and excision of the terminal 10 cm of branches, we observed significant increases in the proportions of hermaphroditic flowers (Figure 4B,D). However, rather than enhancing the total amount of resources accessible to plants, these treatments contributed to modifying the patterns of endogenous resource allocation by mitigating competition from vegetative growth, with the accumulated resources in plants being re‐allocated to the remaining flower buds. Under conditions in which plants have access to ample resources or experience an improvement in resource availability, larger amounts of these resources will be allocated to the more resource‐demanding female function, resulting in a preferential investment in female flowers (Figure 4). Conversely, when plants encounter resource limitation or depletion, a contrasting pattern emerges, characterized by a reduction in the production of hermaphroditic flowers, and a corresponding increase in the proportion of male flowers, which is consistent with the findings reported for *Murdannia simplex* and *Breynia oblongifolia* in response to resource‐constrained conditions (Charnov 1982; Barrett 2013; Finch et al. 2022). Collectively, these findings provide evidence to indicate that the female‐biased floral sex allocation observed in the desert shrub *C. spinosa* is determined to a large extent by the availability of resources.

## Author Contributions

**Yu‐Jie Cai:** data curation (lead), formal analysis (lead), investigation (lead), validation (equal), visualization (lead), writing – original draft (lead). **Lu Yang:** data curation (supporting), investigation (supporting), software (supporting), validation (supporting). **Aysajan Abdusalam:** conceptualization (lead), funding acquisition (lead), methodology (equal), project administration (lead), resources (lead), supervision (lead), writing – review and editing (lead). All authors reviewed and approved the final version of the manuscript.

## Funding

This work was supported by National Natural Science Foundation of China (Grant Number 32460261), the “Tianshan Yingcai”‐Youth Top‐notch Talent Program (Grant Number 2023TSYCCX0026) and the Kashi University Innovation Team Construction project (TD20251125).

## Conflicts of Interest

The authors declare no conflicts of interest.

## Supporting information


**Data S1:** Supporting Information.

**Table S1:** Correlations between aboveground biomass and the numbers of male and hermaphroditic flowers, and Gi values among populations. Values are Spearman rank correlation coefficients (ρ). *Correlation is significant at the 0.05 level (two‐tailed). **Correlation is significant at the 0.01 level (two‐tailed).

## Data Availability

The raw data that support the findings of this study are available in the Supporting Information S1 of this article (Dataset). All other data are included in the main manuscript and its supporting Information S1.
