# Supplementary material for: Resource Availability Shaped the Female‐Biased Floral Sex Allocation With Plant Size in Andromonoecious Capparis spinosa in Desert
Source: Ecol Evol. 2026 Nov 2;16(11):e74605. doi: 10.1002/ece3.74605 (PMC13727282; doi:10.1002/ece3.74605)
Supplement: Supplementary file 1 [file ECE3-16-e74605-s002.docx]

**Table S1:** Correlations between aboveground biomass and the numbers of male and hermaphroditic flowers, and Gi values among populations.

| Population |  | Number of male flowers | Number of hermaphroditic flowers | Gi Value |
| --- | --- | --- | --- | --- |
| Jwz | Aboveground biomass (g) | 0.699** | 0.727** | 0.315* |
| Bjx | Aboveground biomass (g) | 0.877** | 0.865** | 0.353* |
| Ly | Aboveground biomass (g) | 0.774** | 0.866** | 0.367* |
| Smy | Aboveground biomass (g) | 0.839** | 0.938** | 0.261* |
| Ahq | Aboveground biomass (g) | 0.800** | 0.895** | 0.338* |
| Hbq | Aboveground biomass (g) | 0.768** | 0.858** | 0.278* |
| Wq | Aboveground biomass (g) | 0.714** | 0.803** | 0.291* |
| Akt | Aboveground biomass (g) | 0.502** | 0.793** | 0.314* |
| Tx | Aboveground biomass (g) | 0.731** | 0.635** | 0.339** |
| Total | Aboveground biomass (g) | 0.719** | 0.787** | 0.26** |

Note:values are Spearman rank correlation coefficients (ρ).

* Correlation is significant at the 0.05 level (two-tailed).

** Correlation is significant at the 0.01 level (two-tailed).
